# Adaptive Reprogramming During Early Seed Germination Requires Temporarily Enhanced Fermentation-A Critical Role for Alternative Oxidase Regulation That Concerns Also Microbiota Effectiveness

**DOI:** 10.3389/fpls.2021.686274

**Published:** 2021-10-01

**Authors:** Revuru Bharadwaj, Carlos Noceda, Gunasekharan Mohanapriya, Sarma Rajeev Kumar, Karine Leitão Lima Thiers, José Hélio Costa, Elisete Santos Macedo, Aprajita Kumari, Kapuganti Jagadis Gupta, Shivani Srivastava, Alok Adholeya, Manuela Oliveira, Isabel Velada, Debabrata Sircar, Ramalingam Sathishkumar, Birgit Arnholdt-Schmitt

**Affiliations:** ^1^Plant Genetic Engineering Laboratory, Department of Biotechnology, Bharathiar University, Coimbatore, India; ^2^Non-Institutional Competence Focus (NICFocus) ‘Functional Cell Reprogramming and Organism Plasticity’ (FunCROP), coordinated from Foros de Vale de Figueira, Alentejo, Portugal; ^3^Cell and Molecular Biology of Plants (BIOCEMP)/Industrial Biotechnology and Bioproducts, Departamento de Ciencias de la Vida y de la Agricultura, Universidad de las Fuerzas Armadas-ESPE, Sangolquí, Ecuador; ^4^Functional Genomics and Bioinformatics Group, Department of Biochemistry and Molecular Biology, Federal University of Ceará, Fortaleza, Brazil; ^5^National Institute of Plant Genome Research, New Delhi, India; ^6^Centre for Mycorrhizal Research, Sustainable Agriculture Division, The Energy and Resources Institute (TERI), TERI Gram, Gurugram, India; ^7^Department of Mathematics and CIMA - Center for Research on Mathematics and Its Applications, Universidade de Évora, Évora, Portugal; ^8^MED—Mediterranean Institute for Agriculture, Environment and Development, Instituto de Investigação e Formação Avançada, Universidade de Évora, Évora, Portugal; ^9^Department of Biotechnology, Indian Institute of Technology Roorkee, Roorkee, India

**Keywords:** seed quality, ROS, Warburg effect, bacterial endophytes and mycorrhizal fungi, organic seeds, biotic stress, *on-farm* seed selection

## Abstract

Plants respond to environmental cues *via* adaptive cell reprogramming that can affect whole plant and ecosystem functionality. Microbiota constitutes part of the inner and outer environment of the plant. This *Umwelt* underlies steady dynamics, due to complex local and global biotic and abiotic changes. Hence, adaptive plant holobiont responses are crucial for continuous metabolic adjustment at the systems level. Plants require oxygen-dependent respiration for energy-dependent adaptive morphology, such as germination, root and shoot growth, and formation of adventitious, clonal, and reproductive organs, fruits, and seeds. Fermentative paths can help in acclimation and, to our view, the role of alternative oxidase (AOX) in coordinating complex metabolic and physiological adjustments is underestimated. Cellular levels of sucrose are an important sensor of environmental stress. We explored the role of exogenous sucrose and its interplay with AOX during early seed germination. We found that sucrose-dependent initiation of fermentation during the first 12 h after imbibition (HAI) was beneficial to germination. However, parallel upregulated *AOX* expression was essential to control negative effects by prolonged sucrose treatment. Early downregulated *AOX* activity until 12 HAI improved germination efficiency in the absence of sucrose but suppressed early germination in its presence. The results also suggest that seeds inoculated with arbuscular mycorrhizal fungi (AMF) can buffer sucrose stress during germination to restore normal respiration more efficiently. Following this approach, we propose a simple method to identify organic seeds and low-cost *on-farm* perspectives for early identifying disease tolerance, predicting plant holobiont behavior, and improving germination. Furthermore, the research strengthens the view that AOX can serve as a powerful functional marker source for seed hologenomes.

## Introduction

Understanding the role of microbiota in adaptive plant robustness is important for crop improvement and developing innovative tools that could allow more efficient plant selection (Arnholdt-Schmitt et al., 2014; Arnholdt-Schmitt et al., 2015, 2018; Nogales et al., 2015). Research on the relevance of endophytic and associated microbiota and usage of microbes as bioinoculants are often hampered by low reproducibility, due to a lack of better understanding of fundamental principles of the plant-microbe interactions (Arnholdt-Schmitt, 2008; Vicente and Arnholdt-Schmitt, 2008; Campos et al., 2015; Mercy et al., 2015, 2017; Bedini et al., 2018; Albornoz et al., 2020) emphasized the need to study mycorrhizal benefits on a case-by-case basis which should consider more holistic and context-dependent views on mycorrhiza functioning at plant family and biome-wide levels. Also, it is widely confirmed that endophyte effects are genotype-specific (Abdelrazek et al., 2020a,b). Furthermore, Durán et al. (2018) identified bacterial endophytes as drivers for soil suppressive take-all disease. Nevertheless, they highlighted that they did not find a relevant correlation between disease suppression and reduced pathogen biomass. In our opinion, these are key observations. They encouraged us to initiate the work on the hypothesis that the competence of individual plant hosts for resilience plays the most critical role for beneficial or non-beneficial plant-microbe interactions, which can be superior to plant families and biome origins.

However, there is a lack of knowledge on traits that aid in (a) early prediction of the plant strength, (b) demonstration of its relevance for plant-microbe interactions, and (c) transformation of such knowledge toward user- and environment-friendly applications for sustainable agriculture. We earnestly aim with the perspective to understand these phenomena and to contribute to the knowledge base toward closing these three gaps.

### Seed Germination/Reprogramming as a Model to Study Plant Adaptive Robustness

The capacity for efficient reprogramming as a trait *per se* is recognized as a marker for adaptive plant robustness (Cardoso and Arnholdt-Schmitt, 2013). Seed germination can serve as an experimental *in vitro* tool to study environmental stress-induced reprogramming and to identify early functional markers and tools for predicting plant performance under field conditions (Mohanapriya et al., 2019). Dry seeds are known to respond upon water imbibition and subsequent penetration of oxygen. Thus, radicle emergence can be seen as an indicator of environmental stress recovery from the dry-to-water imbibed conditions and low-to-high oxygen status.

Efficient seed germination under field conditions is especially required in organic agriculture, where the application of chemical herbicides and pesticides to suppress competitors shall be avoided to support healthy food and feed production and to improve the sustainability of bio-based socioeconomic systems. At the same time, organic agriculture impacts seed quality and the amount of microbiota in seeds (Cope-Selby et al., 2017; Wassermann et al., 2019). Recently, the use of the so-called “organic seeds” vs. conventionally produced seeds is raised as an ethical issue (European Parliament and of the Council, 2018). However, the better quality of organic seeds in terms of their contribution to agriculture sustainability, nutritional quality, and yield performance is under intensive debate (e.g., Bhaskar et al., 2019; Voss-Fels et al., 2019) and requires scientific validation (Simon et al., 2017; Abdelrazek et al., 2020a,b). Appropriate methods and tools are in absolute need to discriminate organic vs. conventional seeds by traits that should allow predicting the superior quality of organic seeds.

### The Complex Role of Sucrose in Adaptive Reprogramming

Cellular reprogramming is an energy intensive phenomenon. Reactive oxygen species (ROS) are known to interact with redox-sensitive protein cysteine thiol groups relevant for energy metabolism and metabolic channeling linked to cell differentiation and cell cycle regulation (Bigarella et al., 2014; Dumont and Rivoal, 2019; Gupta et al., 2020a,b; Pengpeng et al., 2020; Qi et al., 2020). Sugars and sugar phosphates interact with hormone-mediated signal networks to modulate energy metabolism. Auxin-stimulated sugar metabolism is frequently reported (e.g., Zhao et al., 2021); however, only few examples revealed that sucrose can induce new cell programs (Grieb et al., 1994; see in Zavattieri et al., 2010) and also, vice versa, can change auxin metabolism (Lin et al., 2016; Meitzel et al., 2021). In maize, sucrose induced several cell cycle markers during germination than glucose (Lara-Núñez et al., 2017). Downstream of sugars, two important antagonistic protein kinases are involved in energy sensing and physiological adaptation (reviews in Bailey-Serres et al., 2018; Sakr et al., 2018; Schmidt et al., 2018). While sucrose non-fermenting-1-related protein kinase 1 is activated when energy is depleted (Schmidt et al., 2018; Wurzinger et al., 2018; Wang et al., 2020), the target of rapamycin (TOR) is induced under conditions of energy excess to stimulate the cell cycle progression and the cell proliferation (Sangüesa et al., 2019). Sucrose can have various functions: besides its metabolic role, it acts as a signaling component (Baena-González and Hanson, 2017; Sakr et al., 2018), as an osmotic stressor that can disrupt communication within and between cells (Moon et al., 2015), shown to trigger aerobic alcohol fermentation in support of respiration, and biosynthesis of higher molecular weight compounds, such as lipids (Mellema et al., 2002).

### Multifunctional Role of AOX as Switch Between Respiration and Fermentation During the Germination Process

Alcohol fermentation has been found to play a critical role in controlling tissue level pyruvate in plants, thereby, adjusting respiration rates to prevailing cellular energy status (Zabalza et al., 2009). Fan et al. (2020) identified hormone and alcohol degradation pathways that were mostly activated during the early stages of somatic embryogenesis (SE), which is a prominent example of *de novo* programming. Ethanol has been shown to reduce ROS levels and led to high induction of *alternative oxidase* (*AOX*) and *glutathione-S-transferase* transcripts (Nguyen et al., 2017). Transcriptome analyses at 2,4-dichlorophenoxyacetic acid (2,4-D) induced reprogramming indicated that the extent of aerobic fermentation is connected to cell proliferation and is regulated by interacting levels of sucrose and AOX (Costa et al., 2021). Transient upregulation of genes related to alcoholic and lactic acid fermentation is shown to be associated with glycolysis and modified complex stress signaling patterns with enhanced superoxide dismutase (SOD) and decreased transcript levels of nitric oxide (NO) producing *nitrate reductase* (NR). Furthermore, the data signaled activation of cell death-regulating system and arrested cell cycle by reducing alpha-tubulin gene transcription in the earliest step of reprogramming. Considering the generality of these observations, we proposed a reference transcriptome profile to identify virus traits that link to harmful reprogramming (Arnholdt-Schmitt et al., 2021). This approach helped to identify an early trait for combating SARS-CoV-2 that covers ROS/reactive nitrogen species (RNS) balancing, aerobic fermentation regulation, and cell cycle control (Costa et al., 2021).

In seeds, fermentation and alternative respiration (AR) are dominating metabolic reactions (Arnholdt-Schmitt et al., 2018; Mohanapriya et al., 2019). During seed germination, structural and functional acclimation of aerobic respiration is central and determines the temperature-dependent efficiency of germination (Bello and Bradford, 2016; Paszkiewicz et al., 2017). Nevertheless, markers for respiration and oxygen consumption were not superior to simple germination tests for predicting the vigor of single seeds (Powell, 2017). However, it is suggested that AR plays the most critical role during germination (Arnholdt-Schmitt et al., 2018; Mohanapriya et al., 2019). This role requires managing ROS/RNS increase and channeling energy and substance flow from fermentation, when carbohydrate storages are released and enzymes get into action (Saleh and Kalodimos, 2017), but the respiration chain is still structurally restricted and overloaded by massively incoming oxygen. AOX is mainly regulated by pyruvate (Millar et al., 1996; Hoefnagel et al., 1997; Hakkaart et al., 2006; Albury et al., 2009; Carré et al., 2011; Selinski et al., 2018) and strikingly, Ito et al. (2020) showed in *Arum* that energy-related metabolic regulation can be determined by temperature-dependent switching between AOX polymorphisms in the binding site for AOX-pyruvate. In this scenario, it might be of interest that AOX is essential in ethylene-induced drought tolerance and mediating autophagy *via* balancing ROS levels (Zhu et al., 2018). Also, thermoinhibition of carrot seed germination could be circumvented by seed priming, which was found to be linked to increased ethylene production at higher temperatures (Nascimento et al., 2013). Ethylene biosynthesis is found to be induced by hydrogen peroxide (H_2_O_2_) and acted positively on germination, independent of auxin-coordinated hormonal crosstalk linked to abscisic acid suppression and gibberellin activation (Wojtyla et al., 2016). During ethylene biosynthesis, cyanide is generated as a by-product of the pathway and probably shifts cytochrome oxidase (COX)-mediated respiration to AR (Siegień and Bogatek, 2006; Machingura et al., 2016). Eckert et al. (2014) stressed that microbiota has developed ethylene-producing pathways to profit during the invasion and to evade defense responses of the host plants. Additionally, Mercy et al. (2017) observed that treating mycorrhiza-infected seedlings with potassium cyanide promoted local arbuscular formation.

### AOX Is a Key Molecule for Cellular Reprogramming: Toward a Perspective

Recently, we identified AOX as the stress level sensing coordinator for auxin inducible metabolic reprogramming by comparing induction of SE and seed germination (Arnholdt-Schmitt et al., 2018; Mohanapriya et al., 2019). Association of AOX to target cell reprogramming is also observed in other systems such as adventitious root development in olive (Santos Macedo et al., 2009; Porfirio et al., 2016) and elicitor-induced hairy roots (Sircar et al., 2012). Furthermore, our group had contributed to novel functional marker strategies by highlighting AOX as a marker across taxonomic borders that includes “shared” *AOX* genes in plant holobionts (Arnholdt-Schmitt, 2005a,b, 2008; Arnholdt-Schmitt et al., 2006; Campos et al., 2015; Mercy et al., 2017; Bedini et al., 2018). Based on the role of AOX in carbohydrate metabolism (Vanlerberghe et al., 1994), our approach has been stimulated to understand the role of fermentation and sugars during plant–mycorrhiza interactions (Mercy et al., 2017; Bedini et al., 2018) and had led to a privately explored patent (Lucic and Mercy, 2014). However, the early phase of reprogramming was not sufficiently considered in that research (Mercy et al., 2017) to drive our core functional marker approach (Arnholdt-Schmitt, 2008; Mercy et al., 2015). Recently, Mohanapriya et al. (2019) observed that arbuscular mycorrhizal fungi (AMF) inoculation in carrot seeds interacted with the AOX-inhibitor salicyl hydroxamic acid (SHAM) and palliated negative SHAM effects on early germination. Also, AMF effects in seeds seemed to be modified by non-culturable microbiota. Integrated *in silico* studies on experimental data revealed that endophytes interact with *AOX* expression in species-, stress-, and developmental-dependent manner. Also, Costa et al. (2021) highlighted the importance of microbiota–plant genotype interaction and its impact on early carrot seed germination which can be modified by SHAM.

**FIGURE 1:**
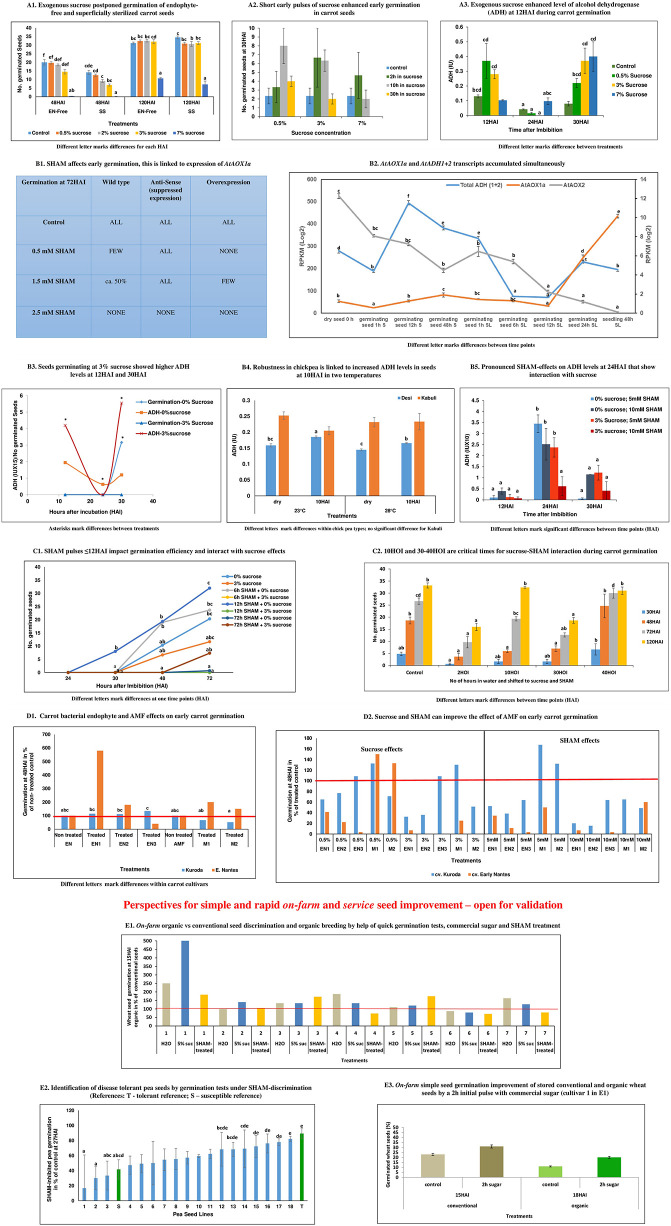
Step-by-step rationale of the perspective. **(A1)** Exogenous sucrose postponed germination of endophyte-free and superficially sterilized carrot seeds: Sucrose inhibited early germination [at 48 h after imbibition (HAI)] depending on increasing sugar concentrations. This trend is the same for endophyte-free seeds that were superficially sterilized. At 120 HAI, the effect of 0.5–3% sucrose could not be noticed anymore, while 7% sucrose inhibited germination for a prolonged time. This observation indicates a critical role of sucrose during the induction of adaptive performance. To confirm, the role of sucrose, in Supplementary Figure 1, the effect of sucrose is shown for auxin-dependent early induction of somatic embryogenesis (SE) as the most studied example of *de novo* programming. It demonstrates that (a) sugar is essential for cell reprogramming since SE induction was not observed at around 45 DAI (days after inoculation) in controls, but only at 2 and 3% sucrose supply and (b) that SE can be optimized with the help of increasing amounts of exogenous sucrose since SE induction efficiency is highest at 3% sucrose (Supplementary Table 1). Cell reprogramming competes with cell division. This is a common insight, which got here validated again through the observed delay in embryonic vs. non-embryonic callus emergence at lower sucrose concentrations. As a general tendency, at higher sucrose levels, less number of seeds showed callus growth, which was later demonstrated to be embryogenic, in comparison to the higher number of seeds with (non-embryogenic) callus growth at lower sucrose levels (Supplementary Figure 1). **(A2)** Short early pulses of sucrose enhanced early germination in carrot seeds: A total of 3% sucrose applied for 2 or 10 h imbibition enhanced early germination for about the same degree compared to the control and longer pulse of 30 h. A lower sucrose concentration of 0.5% had the highest effect only by a longer pulse of 10 h and, at 7% sucrose, a higher effect against the control was only indicated when given as a short pulse of 2 h. This observation was confirmed with a second carrot cultivar in a rapid *on-farm* check by using a ca. 5% solution of commercial sugar (significant) (Supplementary Figure 2). **(A3)** Exogenous sucrose enhanced the level of alcohol-dehydrogenase (ADH) at 12 HAI during carrot germination: At 12 HAI, treatment with 0.5 and 3% sucrose resulted in an increase in ADH activity as compared to the control, while for 7% sucrose no effect is observed. At 24 HAI, the control indicated a decline of ADH values. In the presence of 0.5 and 3% sucrose, this decline is not avoided or might even have been strengthened. However, at 30 HAI, a second phase started, where sucrose enhanced the levels of ADH in a concentration-dependent manner including a positive effect of 7% sucrose. **(B1)** Salicyl hydroxamic acid (SHAM) affects early germination and this links to expression of *AtAOX1a*: In wild-type *Arabidopsis thaliana* seeds, while monitoring germination at 72 HAI, showed that SHAM treatment led to reduced germination rates. This inhibition is dependent on its concentration of 0.5 or 1.5 mM. However, when alternative oxidase (AOX) had been silenced (antisense), SHAM did not affect germination. On the contrary, when AOX was constitutively overexpressed (OE), SHAM indicated stronger inhibition of germination than in the wild type. Nevertheless, the three genotypes germinated with similar efficiency about their respective controls. This latter observation points to the fact that AOX is critically important for germination, if present. However, in case it is not present or activated (antisense) alternative pathways can substitute the functional role of AOX during germination. **(B2)** *AtAOX1a* and *AtADH1* + *2* transcripts accumulated simultaneously: A study on ADH transcript accumulation in wild-type *A. thaliana* confirmed a biphasic activation of *ADH* during germination. A first increase was observed 12 h after stratification (significant), which includes the imbibition of water. The second enhancement occurred from 12 h SL shortly before root emergence was monitored at 24 h SL. In parallel to increased *ADH* transcript accumulation, *AOX1a* transcripts accumulated during both phases, i.e., induction and early initiation of germination. After early induction, *ADH* transcripts showed a high decline (significant) until the end of the dark stratification phase, while *AOX1a* transcript levels remained more stable. During the second phase at the initiation of exponential root length growth in light observed at 48 h SL, *AOX1a* transcript accumulation keeps on enhancing, while the increase of *ADH* transcripts stopped at that time point. This is also indicated in the first phase. *AOX2* transcript accumulation is differentially regulated in comparison to *AOX1a* and showed continuous downregulation during the whole period, which appeared to be stronger in the SL phase. **(B3)** Seeds germinating at 3% sucrose showed higher ADH levels at 12 and 30 HAI: During early germination of carrot seeds, ADH levels follow a parable, when monitored between 12 and 30 HAI. This is observed in control seeds and seeds germinating at 3% sucrose. Nevertheless, suppressed germination at 3% sucrose is linked to higher levels of ADH at 12 and 30 HAI. This means the more efficient germination in control seeds is linked at these two-time points to lower levels of ADH. Under both conditions, in the absence of exogenous sucrose and at 3% sucrose, 24 HAI displayed a turning point with the lowest ADH activity levels. However, ADH activity at 24 HAI is higher in controls (significant) than under conditions of sucrose-supplementation. **(B4)** Early chickpea plant vigor is critical for plant productivity under terminal drought conditions (Sivasakthi et al., 2017). From the two principle chickpea types, Desi and Kabuli, vast field experience has shown that Desi is superior in terms of multistress tolerance and yield performance (Purushothaman et al., 2014). In former research, we could discriminate both types at 10 HAI by a lower oxycaloric equivalent (Rq/RCO_2_; calorespirometric ratio) value due to differential carbon use and, thus, predict *a posteriori* better yield stability of Desi (Mohanapriya et al., 2019). In this study, we show that Desi increased the level of ADH at 10 HAI during germination (significant at 23 and 28°C), while this is not seen in Kabuli. The reached level of ADH is higher at 23°C than at 28°C. **(B5)** Pronounced SHAM effects on ADH levels at 24 HAI that show interaction with sucrose: During the germination of carrot seeds, the most pronounced effect of SHAM treatment on ADH levels is observed at 24 HAI. At that time point, SHAM stimulated ADH levels compared to levels observed at 12 and 30 HAI. This happened independently of the presence of sucrose (3%). However, under both conditions, 5 mM SHAM showed a stronger stimulating effect on ADH levels (significant) at 24 HAI than 10 mM SHAM. But the level of SHAM-enhanced ADH is higher at both tested concentrations of SHAM when sucrose is not present. On the contrary, at time points 12 and 30 HAI, a higher ADH level in the 0% sucrose controls is associated with the higher concentration of 10 mM SHAM versus 5 mM SHAM. In the presence of 3% sucrose, ADH activity is at any time point higher at 5 mM SHAM than at 10 mM SHAM. Together, these observations point to the importance of differential AOX activity regulation for optimized germination during all three time points independently of the presence or absence of exogenous sucrose. **(C1)** SHAM pulses ≤12 HAI impact germination efficiency and interact with sucrose effects: In control seeds, short pulses of SHAM (10 mM) until 12 HAI enhanced germination efficiency and were more effective than pulses until 6 HAI. However, prolonged SHAM treatment of 72 HAI suppressed early germination. In contrast, at 3% exogenous sucrose, early germination efficiency is reduced against 0% sucrose controls (confirming panel **A1**) and SHAM pulses until 6 and 12 HAI led to complete suppression of early germination. However, from 48 HAI onward to 72 HAI, continuous SHAM treatment in the presence of 3% sucrose increased germination, but along with 0% sugar continuous SHAM suppressed germination also at 72 HAI. Collectively, these results show that plastic AOX regulation is critical for the timing of germination in controls and under conditions of sucrose supplementation. **(C2)** 10 and 30–40 HAI are critical times for sucrose–SHAM interaction during carrot germination: A total of 10 h of previous water imbibition reduced the strong negative effects of the combination of exogenous sucrose (3%) and SHAM (5 mM) on germination efficiency that was observed at only 2 h of previous water imbibition (significant). Also, during the phase of initiated root emergence at 30 Hours of imbibition (HOI), a transfer from water to media supplemented with sucrose and SHAM suppressed germination (significant). Water imbibition until 40 h before transfer to sucrose- and SHAM-containing media relieved and even supported germination when monitored at 30 and 48 HAI (significant). However, this increase in germination efficiency seemed to be restricted from 72 HAI onward (significant). **(D)** Sucrose and SHAM can improve the effect of arbuscular mycorrhizal fungi (AMF) on early germination: In panel **D1**, it is shown that carrot seeds treated with native endophytes (EN1—endophyte 1; EN2—endophyte 2; EN3—endophyte 3; isolated from cv. Kuroda) tend to improve early germination at 48 HAI in both the cultivars (not seen for EN3 in cv. Early Nantes). Exogenous sucrose had differential effects depending on endophyte and cultivar (Panel **D2**), but in no case does sucrose enhances early germination rates compared to the respective endophyte-treated controls (as shown also in Supplementary Table 2). However, SHAM treatment (Panel **D2**) reduced early germination against endophyte-treated controls in all cases (as shown also in Supplementary Table 2). In a separate trial, two AMF species (*R. irregularis*—M1 and *R. proliferus*—M2) were tested and acted negatively on germination in cv. Kuroda, but positively in slowly germinating seeds of cv. Early Nantes (Panel **D1**). Nevertheless, the effect of M1 on early germination could be improved in cv. Kuroda by 0.5 and 3% sucrose (Panel **D2**). However, this was not seen for M2. In the better germinating cv. Kuroda, the lower concentration of 5 mM SHAM (Panel **D2**) improved the effect of both mycorrhiza species on early germination. In later germinating seeds of cv. Early Nantes, 0.5% sucrose improved the already positive effect on germination (Panel **D1**) of *Rhizophagus* species M1 (Panel **D2**). In this cultivar, SHAM decreased the germination rate to the level of the untreated controls (Supplementary Table 2). **(E1)** *On-farm* organic vs. conventional seed discrimination and organic breeding with the help of quick germination tests, commercial sugar, and SHAM treatment: Seeds from six of seven winter wheat cultivars originated from organic agricultural management could be discriminated at 15 HAI through better germination against conventionally produced seeds when germinated in 5% sugar solution. In water, seeds of only four cultivars showed better germination than organic seeds. When conventionally produced material is compared, seeds of the first cultivar showed poor germination. This was much more pronounced when tested in a 5% sugar solution instead of water. Seeds of the second cultivar demonstrated the highest germination rates among all tested cultivars (as shown in Supplementary Figure 4). This was observed for seeds originating from both agricultural conditions, although higher germination in 5% sugar solution indicated the presence of microbiota (Panel **E1**). In contrast to all other cultivars, seeds of the second cultivar did not differ in germination rates for organic vs. conventional production under SHAM treatment when compared to the water control (as shown in also Supplementary Figure 4). This signals already low levels of AOX at 15 HAI for this cultivar no matter from which agricultural management system seeds originated. Overall, these observations indicate the interplay between plant genotype, sugar, and AOX activity that impacts differential germination capacities between organic and conventional seeds. **(E2)** Identification of disease tolerant pea seeds by germination tests under SHAM discrimination (T-tolerant reference; S-susceptible reference): Pea lines with differential degrees of root rot disease susceptibility could be ranked by employing SHAM inhibition. The most tolerant line (T) showed the lowest degree of SHAM-related inhibition of germination monitored at 27 HAI. This indicates the reasonability of germination tests under SHAM discrimination for the selection of seed vigor and plant robustness. **(E3)** *On-farm* simple seed germination improvement of stored conventional and organic wheat seeds by a 2 h initial pulse with commercial sugar (first cultivar in 1E1): This figure demonstrates the general potential of improving early germination through a short pulse of sugar its validity across species (in this study winter wheat, but also refer for carrot in Panel **A2** and Supplementary Figure 2), agricultural management practices and also related to the aging of seeds.

In our earlier work in Mohanapriya et al. (2019), we demonstrated successful prediction of oxycaloric equivalents from germinating seeds at 10 HAI. The present perspective questions the metabolic nature of AOX coordination and provides deeper phenotyping during germination of endophyte-free and microbiota-inoculated seeds focused at early times around 12 HAI. Figure 1 demonstrates the step-by-step rationale of fundamental insights and deduced practical strategies (methodology of experiments is provided in Supplementary File 1).

**In our findings**, we observed that **(a)** during *Arabidopsis thaliana* seed germination *alcohol dehydrogenase* (ADH) transcript levels were increased at 12 h after seed stratification (SL) in water followed by a decline, and the increase in *ADH* transcript levels is in general accompanied by increased *AOX1a* transcript accumulation (Figure 1B2). **(b)** In agreement with **(a)**, germinating carrot seeds displayed a higher level of ADH activity at 12 HAI than 24 HAI. In the presence of 3% sucrose, this level was further enhanced (Figures 1A3,B3). **(c)** Two hours short pulses of sucrose before water imbibition enhanced early germination in seeds of two different species, viz., carrot and wheat (Figures 1A2,E3 and Supplementary Figure 2). Additionally, in carrots, we showed that the effectiveness of such early sugar pulse was dependent on sucrose concentration. A short pulse could be substituted by a longer pulse at a lower concentration of sucrose (Figure 1A2). **(d)** On the contrary, SHAM treatment until 6 and 12 HAI suppressed germination in the presence of 3% sucrose. However, it favored early germination in the absence of sucrose (Figure 1C1).

**(e)** Three carrot native bacterial endophytes (EN1, EN2, and EN3) were used for carrot seed inoculations with two cultivars (cv. Kuroda, cv. Early Nantes) and showed a tendency to improve germination (Figure 1D1). However, a positive effect is dependent on cultivar-endophyte interaction. SHAM treatment reduced the early germination percentage of endophyte-treated seeds against the respective endophyte-treated controls. This was observed in all cases though to a different degree of inhibition (Figure 1D2 and Supplementary Table 2). **(f)** Sucrose has displayed different impacts on endophyte-mediated effects on germination and is dependent on cultivar and endophytes. However, in no case did endophytes improved germination of sucrose-treated seeds when compared with endophyte-treated controls without sucrose (Figure 1D2 and Supplementary Table 2). **(g)** In a good germinating carrot cultivar (cv. Kuroda), the two selected *Rhizophagus* species (*Rhizophagus irregularis* and *Rhizophagus proliferus*) acted negatively on early germination, while in a delayed germinating carrot cultivar (cv. Early Nantes), both *Rhizophagus* species acted positively (Figure 1D1 and Supplementary Table 2). In both cultivars, sucrose could improve *Rhizophagus* effects on early germination to higher levels than the AMF-treated controls. However, this is dependent on cultivar-species interaction. In the presence of sucrose, *R. irregularis* (M1) improved germination of both cultivars compared to M1-treated control seeds in the absence of sucrose (Figure 1D2). **(h)** In addition, at lower concentrations of SHAM (5 mM), early germination could be improved to higher levels as compared to the AMF-treated controls (Figure 1D2), but this is observed only in the cv. Kuroda variety, which did not show positive AMF effects against non-AMF-treated controls (Figure 1D1 and Supplementary Table 2).

**FIGURE 2:**
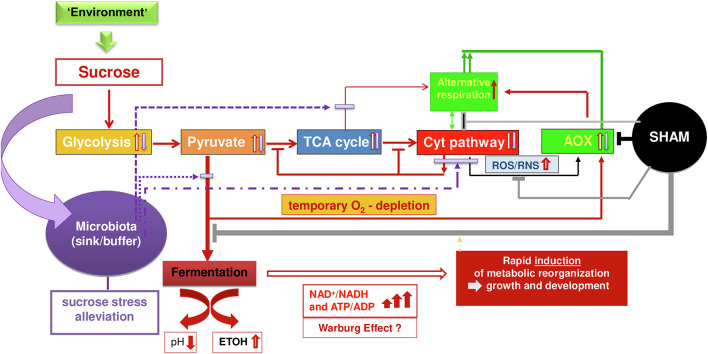
A simplified scheme on hypothesis and conceptualization for working out metabolic principles on dynamic cell reprogram functioning (details explained in the text).

In Figure 2, we present a simplified scheme that summarizes our conclusion based on wet-lab experiments, state-of-the-art knowledge, and our hypothetical inferences related to the dynamic metabolic interplay between sucrose, aerobic fermentation, COX-mediated respiration, AOX regulation/AR, and microbiota on cell reprogram functioning. In this scheme, we separated AOX as a macromolecule (gene/protein) from its functional pathway, AR, to highlight the outstanding position of AOX as the key and only enzyme of the pathway that, if present in an organism, is recognized to provide a central metabolism-coordinating function for efficient survival (Mohanapriya et al., 2019; Arnholdt-Schmitt et al., 2021; Costa et al., 2021). We consider that under development- and/or environment-induced conditions of rapid sucrose increase, the COX pathway is stimulated *via* enhanced glycolysis, pyruvate production, and increased tricarboxylic acid (TCA) cycle, in a way that the respiratory chain can get overloaded by electrons followed by enhanced ROS/RNS levels and, on the other hand, restricted due to rapidly consumed oxygen and/or yet low numbers of functional mitochondria concerning the presence of oxygen during germination. In response, aerobic alcoholic and lactic fermentation are stimulated (refer points a, b, and c; Costa et al., 2021). At the same time, AOX is activated [refer point d and in Mohanapriya et al. (2019), Costa et al. (2021)] mainly through *AOX* gene sequence-dependent pyruvate regulation and ROS/RNS.

Depending on stress level and the amount of sucrose and duration of the situation of high sugar-level, anaerobic glycolysis can reach high turnover during cell reprogramming and high levels of adenosine triphosphate production even corresponding to the Warburg effect. This latter hypothesis is supported by parallel research on auxin-induced callus growth (Costa et al., 2021), where we observed a rapid increase in *ADH1* transcripts of 1,777% and a parallel increase in *LDH* (*Lactate dehydrogenase*) transcripts of 346%. Warburg effects are increasingly recognized also in human systems (Kutschera et al., 2020; Melkonian and Schury, 2020) as being part of normal physiology. However, in plants, they are studied still more concerning photosynthesis (Kutschera et al., 2020) and anaerobic conditions are best explored under flooding conditions and are related to anaerobic tolerance in rice (Narsai et al., 2017). It is shown that AOX plays a beneficial role under low oxygen and especially during reoxygenation (Jayawardhane et al., 2020).

Under increased sucrose, fermentation can escape feedback downregulation with the help of enhanced AR, since AOX-transferred electrons enable the continuation of the TCA cycle for metabolic reorganization though with relatively less energy efficiency. Thus, fermentation and AOX are complementing each other to maintain metabolic and energetic homeostasis thereby avoiding inefficient situations when the respiration chain is overloaded with oxygen availability. As soon as oxidative stress gets sufficiently diminished at equilibrated oxygen availability in the COX pathway, AOX will be downregulated and normal respiration will reach priority again for driving growth and development. Fermentation and AOX downregulation will again be regulated in adaptation to sucrose- and COX-mediated respiration-transmitted conditions embedded in adaptive hormonal crosstalk and overall complex cellular and apoplastic network signaling. Thus, rapid downregulation indicates efficient adaptation of COX-mediated respiration, a dynamic trait appropriate to mark seed vigor (Mohanapriya et al., 2019).

Sucrose can improve early germination of *Rhizophagus*-treated seeds (as shown in point g) while non-AMF-treated seeds respond upon sucrose typically with a delay in germination (as shown in Figure 1A1). This suggests that AMF and its associated bacteria (Pandit et al., under review) can alleviate or buffer the negative effects of sucrose on germination to relevant degrees by providing an additional sink. This is not indicated with the three tested endophytes (f). Also, early germination of endophyte-treated seeds is reduced at 48 HAI through the continuous presence of SHAM when compared to endophyte-treated controls (e). On the contrary, when seeds from the cv. Kuroda were inoculated with *Rhizophagus*, SHAM treatment (5 mM) could improve early germination to higher levels than observed in AMF-treated controls. This observation is in agreement with the palliating effects observed by *Scutellospora calospora* on negative SHAM effects on carrot germination by using the same cultivar (Mohanapriya et al., 2019). In an overall assessment, it is inferred that AMF treatment might improve early germination by alleviating stress-induced by rapid sucrose excess through two mechanisms: providing an additional sink for sucrose and supplying an enhanced capacity and/or engagement of AR. *Rhizophagus* spores were shown to be a rich source for polymorphic *AOX* gene sequences (Campos et al., 2015). We believe that there could be an operation of two separate mechanisms since we observed differential effects on early germination of M1-treated seeds upon SHAM treatment in the two selected cultivars (Figure 1D). However, M1-treated seeds of both cultivars showed improvement in early germination when sucrose was provided (Figure 1D). We tend to interpret that the isolated native carrot endophytes were already well integrated into the internal host cell habitat. Thus, their reinoculation tended to influence early germination positively, but could not provide a striking new advantage or disadvantage when sucrose is enhanced or SHAM treatment reduced the level of AR. However, we reported that endophytes modulate *AOX* transcripts in species-, stress-, and development-dependent manner, and endophytes could have modified the effect of AMF inoculation on seed germination efficiency (Mohanapriya et al., 2019).

## Outlook

The observations offer new perspectives for low-cost prediction of plant holobiont behavior of seeds and for providing simple and rapid *on-farm* support toward sustainable agriculture. We propose three tools for validation:

(A) Seed selection with the help of short germination tests under SHAM discrimination. This tool provides modalities to identify seeds with higher seed vigor, general adaptive plant robustness, and superior internal seed quality related to the content of secondary metabolites (Figures 1E1,E2 and Supplementary Figures 3, 4).

(B) Discrimination of organic vs. conventionally produced seeds with the help of short duration germination tests in water solutions with 5% commercial sugar (Figure 1E1).

(C) Germination improvement by 2-h pulses of commercial sugar (Figures 1A1,E3 and Supplementary Figure 1).

Furthermore, we encourage developing novel tests for AMF functionality in germinating seeds in the presence of sucrose. This approach targets compatibility between selected plants and AMF strains to support plant holobiont plasticity.

Our results suggest that polymorphic *AOX* gene sequences of symbiotic partners can impact plant–AMF compatibility. Therefore, we want to accomplish wider screening of major *AOX* polymorphisms in species-specific target cells for evaluating plant performance (Abe et al. 2002; Arnholdt-Schmitt, 2015; Nogales et al., 2016) and in AMF sources (Arnholdt-Schmitt, 2008; Vicente and Arnholdt-Schmitt, 2008; Campos et al., 2015). Such a strategy needs to also include near neighboring polymorphisms in conserved functional sites that can discriminate differentially regulated *AOX1* and *AOX2* (Costa et al., 2009). This approach would include a screening of compatible *AOX* polymorphisms from both partners in the proposed functional tests to identify the best plant–AMF combinations.

We hypothesize that the observed integration of bacterial endophytes into host plants with similar sensitivity against SHAM effects might point to synchronized AOX regulation in plant holobionts. Into this derivation would fit that we observed the same tendency of inhibiting sucrose effects on endophyte-free and superficially sterilized seeds (Figure 1A1), which we noticed also for SE induction (unpublished). Vicente et al. (2015) highlighted a “provocative” lack of interest in bacterial *AOX*. They anticipated that bacteria-harboring *AOX* could facilitate adaptation to extreme conditions, which could also be of interest when thinking about plant endophytes and AMF-associated bacteria (Pandit et al., under review).

This present perspective is complementing Mohanapriya et al. (2019) and Costa et al. (2021). Joining the central figures of these publications is thought of as one teaching tool that can help to explain a straightforward way from fundamental interdisciplinary research to the application that might support sustainable socioeconomies because of the diversity of emergent environmental changes.

## Dedication

The authors want to dedicate their work to FREEDOM and ETHICS.

## Data Availability Statement

The original contributions presented in the study are included in the article/Supplementary Material, further inquiries can be directed to the corresponding authors.

## Author Contributions

RB performed lab analyses on carrot germination, endophyte isolation, and inoculation trials related to Figures 1A1–3,B3,4,5, C1,2,D. JHC coordinated transcriptome analyses supported by KT. JHC, RS, and CN discussed initially the approach of this manuscript with BA-S. GM carried out work on Supplementary Figure 1 and Supplementary Table 1. SS was responsible for AMF inoculation trials under the head of AA and EM performed pea studies for Figure 1E2 under the responsibility of BA-S. Under the supervision of KG and EM together with AK performed germination analyses of transgenic *Arabidopsis*, and AK carried out the ADH analyses on chickpea. BA-S performed *on-farm* analyses (Figures 1E1,E3). CN was responsible for statistics and was in part supported by MO. RB and IV helped BA-S in the literature search. DS contributed with Supplementary Figure 3. BA-S initiated the scientific approach, coordinated overall research, and discussion, and wrote the manuscript. All the co-authors commented on research and manuscript during its development and agreed to manuscript submission. RB organized manuscript submission.

## Conflict of Interest

The authors declare that the research was conducted in the absence of any commercial or financial relationships that could be construed as a potential conflict of interest.

## Publisher’s Note

All claims expressed in this article are solely those of the authors and do not necessarily represent those of their affiliated organizations, or those of the publisher, the editors and the reviewers. Any product that may be evaluated in this article, or claim that may be made by its manufacturer, is not guaranteed or endorsed by the publisher.

## Supplementary Material

Supplementary Figure 1 Exogenous sucrose delayed callus emergence and was necessary for SE.

Supplementary Figure 2 A total of 2-h pulse with commercial sugar improved carrot germination efficiency monitored at 40 and 50 HAI.

Supplementary Figure 3 Effect of SHAM treatment on accumulation of soluble and wall-bound phenolics **(A)** and flavonoids and lignin **(B)** in elicitor-treated hairy roots of *Daucus carota*. Values obtained in only elicitor-treated root were considered as 100% and results were expressed in terms of percentage of maximum. The terms E and NE in the x-axis legend denote -with and -without elicitor, respectively. ^∗^ Soluble phenolics. Values are mean of three independent experiments ± SD.

Supplementary Figure 4 Rapid germination check of organic and conventional seeds from seven cultivars in water (control) or under SHAM (5 mM) treatment.

Supplementary Table 1 Effect of exogenous sucrose concentration on carrot SE callus induction.

Supplementary Table 2 Microbiota effect on the carrot seed germination at different sucrose and SHAM concentrations.

## References

[B1] Abdelrazek S. Choudhari S. Thimmapuram J. Simon P. Colley M. Mengiste T. (2020a). Changes in the core endophytic mycobiome of carrot taproots in response to crop management and genotype. *Sci. Rep.* 10:13685. 10.1038/s41598-020-70683-x 32792547 PMC7426841

[B2] Abdelrazek S. Simon P. Colley M. Mengiste T. Hoagland L. (2020b). Crop management system and carrot genotype affect endophyte composition and *Alternaria dauci* suppression. *PLoS One* 15:e0233783. 10.1371/journal.pone.0233783 32497087 PMC7272071

[B3] Abe F. Saito K. Miura K. Toriyama K. (2002). A single nucleotide polymorphism in the alternative oxidase gene among rice varieties differing in low temperature tolerance. *FEBS Lett.* 527 181–185. 10.1016/s0014-5793(02)03218-0 12220657

[B4] Albornoz F. E. Orchard S. Standish R. J. Dickie I. A. Bending G. D. Hilton S. (2020). Evidence for niche differentiation in the environmental responses of co-occurring mucoromycotinian fine root endophytes and glomeromycotinian arbuscular mycorrhizal fungi. *Microb. Ecol.* 81 864–873. 10.1007/s00248-020-01628-0 33145650

[B5] Albury M. S. Elliott C. Moore A. L. (2009). Towards a structural elucidation of the alternative oxidase in plants. *Physiol. Plant* 137 316–327. 10.1111/j.1399-3054.2009.01270.x 19719482

[B6] Arnholdt-Schmitt B. (2005a). Efficient cell reprogramming as a target for functional-marker strategies? Towards new perspectives in applied plant-nutrition research. *J. Plant Nutr. Soil Sci.* 168 617–624.

[B7] Arnholdt-Schmitt B. (2005b). Functional markers and a ‘systemic strategy’: convergency between plant breeding, plant nutrition and molecular biology. *Plant Physiol. Biochem.* 43 817–820. 10.1016/j.plaphy.2005.08.011 16289946

[B8] Arnholdt-Schmitt B. (2008). “A novel gene-candidate approach of socio-economic interest?–breeding on efficient plant genotype - mycorrhiza interaction,” in *Proceedings of the COST 870 Meeting from Production to Application of Arbuscular Mycorrhizal Fungi in Agricultural Systems: a Multidisciplinary Approach*, (Denmark: Department of Integrated Pest Management).

[B9] Arnholdt-Schmitt B. Costa J. H. de Melo D. F. (2006). AOX–a functional marker for efficient cell reprogramming under stress? *Trends Plant Sci.* 11 281–287. 10.1016/j.tplants.2006.05.001 16713324

[B10] Arnholdt-Schmitt B. (2015). “From AOX diversity to functional marker development,” in *Alternative Respiratory Pathways in Higher Plants*, eds Gupta K. J. MuR L. A. J. NeelwarnE B. (Oxford: John Wiley and Sons), 233–243.

[B11] Arnholdt-Schmitt B. Hansen L. D. Nogales A. (2015). Calorespirometry, oxygen isotope analysis and functional-marker assisted selection (‘CalOxy-FMAS’) for genotype screening: a novel concept and tool kit for predicting stable plant growth performance and functional marker identification. *Brief. Funct. Genomics.* 15 10–15. 10.1093/bfgp/elv008 25818699

[B12] Arnholdt-Schmitt B. Mohanapriya G. Sathishkumar R. Macedo E. S. Costa J. H. (2018). “Predicting biomass production from plant robustness and germination efficiency by calorespirometry,” in *Biofuels: Greenhouse Gas Mitigation and Global Warming. Next Generation Biofuels and Role of Biotechnology*, eds Kumar A. Ogita S. Yau Y. (New Delhi: Springer Nature), 81–94.

[B13] Arnholdt-Schmitt B. Valadas V. Doering M. (2014). Functional marker development is challenged by the ubiquity of endophytes – a practical perspective. *Brief. Funct. Genom.* 15 16–21. 10.1093/bfgp/elu049 25526729

[B14] Arnholdt-Schmitt B. Mohanapriya G. Bharadwaj R. Noceda C. Macedo E. S. Sathishkumar R. (2021). From plant survival under severe stress to anti-viral human defense– a perspective that calls for common efforts. *Front. Immunol.* 12:673723. 10.3389/fimmu.2021.673723 34211468 PMC8240590

[B15] Baena-González E. Hanson J. (2017). Shaping plant development through the SnRK1-TOR metabolic regulators. *Curr. Opin. Plant Biol.* 35 152–157. 10.1016/j.pbi.2016.12.004 28027512

[B16] Bailey-Serres J. Pierik R. Ruban A. Wingler A. (2018). The dynamic plant: capture, transformation, and management of energy. *Plant Physiol.* 176 961–966. 10.1104/pp.18.00041 29438068 PMC5813544

[B17] Bedini A. Mercy L. Schneider C. Franken P. Lucic-Mercy E. (2018). Unraveling the initial plant hormone signaling, metabolic mechanisms and plant defense triggering the endomycorrhizal symbiosis behavior. *Front. Plant Sci.* 9:1800. 10.3389/fpls.2018.01800 30619390 PMC6304697

[B18] Bello P. Bradford K. (2016). Single-seed oxygen consumption measurements and population-based threshold models link respiration and germination rates under diverse conditions. *Seed Sci. Res.* 26 199–221. 10.1017/S0960258516000179

[B19] Bhaskar A. V. V. Baresel J. P. Weedon O. Finckh M. R. (2019). Effects of ten years organic and conventional farming on early seedling traits of evolving winter wheat composite cross populations. *Sci. Rep.* 9:9053. 10.1038/s41598-019-45300-1 31227728 PMC6588703

[B20] Bigarella C. L. Liang R. Ghaffari S. (2014). Stem cells and the impact of ROS signaling. *Development* 141 4206–4218. 10.1242/dev.107086 25371358 PMC4302918

[B21] Boratyn G. M. Thierry-Mieg J. Thierry-Mieg D. Busby B. Madden T. L. (2019). Magic-BLAST, an accurate RNA-seq aligner for long and short reads. *BMC Bioinformatics* 20:405. 10.1186/s12859-019-2996-x 31345161 PMC6659269

[B22] Campos C. Cardoso H. Nogales A. Svensson J. Lopez-Ráez J. A. Pozo M. J. (2015). Intra and inter-spore variability in rhizophagus irregularis AOX gene. *PLoS One* 10:e0142339. 10.1371/journal.pone.0142339 26540237 PMC4634980

[B23] Cardoso H. G. Arnholdt-Schmitt B. (2013). “Functional marker development across species,” in *Selected Traits in Diagnostics in Plant Breeding*, eds Lübberstedt T. Varshney R. K. (Netherlands: Springer), 467–515.

[B24] Carré J. E. Affourtit C. Moore A. L. (2011). Interaction of purified alternative oxidase from thermogenic Arum maculatum with pyruvate. *FEBS Lett.* 585 397–401. 10.1016/j.febslet.2010.12.026 21187094

[B25] Cope-Selby N. Cookson A. Squance M. Donnison I. Flavell R. Farrar K. (2017). Endophytic bacteria in miscanthus seed: implications for germination, vertical inheritance of endophytes, plant evolution and breeding. *GCB Bioenergy* 9 57–77.

[B26] Costa J. H. Cardoso H. G. Campos M. D. Zavattieri A. Frederico A. M. de Melo D. F. (2009). Daucus carota L.—an old model for cell reprogramming gains new importancethrough a novel expansion pattern of alternative oxidase (AOX) genes. *Plant Physiol. Biochem.* 47 753–759. 19372042 10.1016/j.plaphy.2009.03.011

[B27] Costa J. H. Mohanapriya G. Bharadwaj R. Noceda C. Thiers K. L. L. Shahid A. (2021). ROS/RNS balancing, aerobic fermentation regulation and cell cycle control – a complex early trait (‘CoV-MAC-TED’) for combating SARS-CoV-2-induced cell reprogramming. *Front. Immunol.* 12:673692. 10.3389/fimmu.2021.673692 34305903 PMC8293103

[B28] Doner L. W. Becard G. (1991). Solubilization of gellan gels by chelation of cations. *Biotechnol. Tech.* 5 25–28.

[B29] Dumont S. Rivoal J. (2019). Consequences of oxidative stress on plant glycolytic and respiratory metabolism. *Front. Plant Sci.* 10:166. 10.3389/fpls.2019.00166 30833954 PMC6387960

[B30] Durán P. Tortella G. Viscardi S. Barra P. J. Carrión V. J. Mora M. L. (2018). Microbial community composition in take-all suppressive soils. *Front. Microbiol.* 9:2198. 10.3389/fmicb.2018.02198 30283421 PMC6156431

[B31] Eckert C. Xu W. Xiong W. Lynch S. Ungerer J. Tao L. (2014). Ethylene-forming enzyme and bioethylene production. *Biotechnol. Biofuels* 7:33. 10.1186/1754-6834-7-33 24589138 PMC3946592

[B32] Fan Y. Yu X. Guo H. Wei J. Guo H. Zhang L. (2020). Dynamic transcriptome analysis reveals uncharacterized complex regulatory pathway underlying dose iba induced embryogenic redifferentiation in cotton. *Int J. Mol. Sci.* 21:426. 10.3390/ijms21020426 31936561 PMC7013799

[B33] Grieb B. Groß U. Pleschka E. Arnholdt-Schmitt B. Neumann K. H. (1994). Embryogenesis of photoautotrophic cell cultures of Daucus carota L. *Plant Cell Tissue Organ. Cult.* 38 115–122.

[B34] Gupta K. J. Hancock J. T. Petrivalsky M. Kolbert Z. Lindermayr C. Durner J. (2020a). Recommendations on terminology and experimental best practice associated with plant nitric oxide research. *New Phytol.* 225 1828–1834. 10.1111/nph.16157 31479520

[B35] Gupta K. J. Kolbert Z. Durner J. Lindermayr C. Corpas F. J. Brouquisse R. (2020b). Regulating the regulator: nitric oxide control of post-translational modifications. *New Phytol.* 227 1319–1325. 32339293 10.1111/nph.16622

[B36] Hakkaart G. A. Dassa E. P. Jacobs H. T. Rustin P. (2006). Allotopic expression of a mitochondrial alternative oxidase confers cyanide resistance to human cell respiration. *EMBO Rep.* 7 341–345. 10.1038/sj.embor.7400601 16322757 PMC1456879

[B37] Hirschauer N. Becker C. (2020). Paradigmenwechsel Warum statistische Signifikanztests abgeschafft werden sollten. *Signifikanztests Forschung Lehre* 6:20.

[B38] Hoefnagel M. Rich P. R. Zhang Q. Wiskich J. T. (1997). Substrate kinetics of the plant mitochondrial alternative oxidase and the effects of pyruvate. *Plant Physiol.* 115 1145–1153. 10.1104/pp.115.3.1145 12223863 PMC158579

[B39] Ito K. Ogata T. Seito T. Umekawa Y. Kakizaki Y. Osada H. (2020). Degradation of mitochondrial alternative oxidase in the appendices of Arum maculatum. *Biochem. J.* 477 3417–3431. 10.1042/BCJ20200515 32856714 PMC7505559

[B40] Jayawardhane J. Cochrane D. W. Vyas P. Bykova N. V. Vanlerberghe G. C. Igamberdiev A. U. (2020). Roles for plant mitochondrial alternative oxidase under normoxia, hypoxia, and reoxygenation conditions. *Front. Plant Sci.* 11:566. 10.3389/fpls.2020.00566 32499803 PMC7243820

[B41] Kagi J. H. Vallee B. L. (1960). The role of zinc in alcohol dehydrogenase. V. The effect of metal-binding agents on thestructure of the yeast alcohol dehydrogenase molecule. *J. Biol. Chem.* 235 3188–3192. 13750715

[B42] Kutschera U. Pieruschka R. Farmer S. Berry J. A. (2020). The Warburg-effects: basic metabolic processes with reference to cancer development and global photosynthesis. *Plant Signal Behav.* 15:1776477. 10.1080/15592324.2020.1776477 32508236 PMC8570714

[B43] Lara-Núñez A. García-Ayala B. B. Garza-Aguilar S. M. Flores-Sánchez J. Sánchez-Camargo V. A. Bravo-Alberto C. E. (2017). Glucose and sucrose differentially modify cell proliferation in maize during germination. *Plant Physiol. Biochem.* 113 20–31. 10.1016/j.plaphy.2017.01.018 28157579

[B44] Lin X. Y. Ye Y. Q. Fan S. K. Jin C. W. Zheng S. J. (2016). Increased sucrose accumulation regulates iron-deficiency responses by promoting auxin signaling in arabidopsis plants. *Plant Physiol.* 170 907–920. 10.1104/pp.15.01598 26644507 PMC4734570

[B45] Lucic E Mercy L. (2014). A method of mycorrhization of plants and use of saccharides in mycorrhization. *Eurpean Patent EP2982241A1.*

[B46] Machingura M. Salomon E. Jez J. M. Ebbs S. D. (2016). The β-cyanoalanine synthase pathway: beyond cyanide detoxification. *Plant Cell Environ.* 39 2329–2341. 10.1111/pce.12755 27116378

[B47] Meitzel T. Radchuk R. McAdam E. L. Thormählen I. Feil R. Munz E. (2021). Trehalose 6-phosphate promotes seed filling by activating auxin biosynthesis. *New Phytol.* 229 1553–1565. 10.1111/nph.16956 32984971

[B48] Melkonian E. A. Schury M. P. (2020). *Biochemistry, Anaerobic Glycolysis.* Available online at: https://www.ncbi.nlm.nih.gov/books/NBK546695/ (accessed Jan 2021). 31536301

[B49] Mellema S. Eichenberger W. Rawyler A. Suter M. Tadege M. Kuhlemeier C. (2002). The ethanolic fermentation pathway supports respiration and lipid biosynthesis in tobacco pollen. *Plant J.* 30 329–336. 10.1046/j.1365-313x.2002.01293.x 12000680

[B50] Mercy L. Lucic-Mercy E. Nogales A. Poghosyan A. Schneider C. Arnholdt-Schmitt B. (2017). A functional approach towards understanding the role of the mitochondrial respiratory chain in an endomycorrhizal symbiosis. *Front. Plant Sci.* 8:417. 10.3389/fpls.2017.00417 28424712 PMC5371606

[B51] Mercy L. Svensson J. T. Lucic E. Cardoso H. G. Nogales A. Döring M. (2015). “AOX gene diversity in arbuscular mycorrhizal fungi (AMF) products – a special challenge,” in *Alternative Respiratory Pathways in Higher Plants*, eds Gupta K. J. Mur L. Neelwarne B. (Oxford: John Wiley and Sons Inc), 305–310.

[B52] Millar A. H. Hoefnagel M. Day D. A. Wiskich J. T. (1996). Specificity of the organic acid activation of alternative oxidase in plant mitochondria. *Plant Physiol.* 111 613–618. 10.1104/pp.111.2.613 12226315 PMC157873

[B53] Mohanapriya G. Bharadwaj R. Noceda C. Costa J. H. Kumar S. R. Sathishkumar R. (2019). Alternative Oxidase (AOX) senses stress levels to coordinate auxin-induced reprogramming from seed germination to somatic embryogenesis—a role relevant for seed vigor prediction and plant robustness. *Front. Plant Sci.* 10:1134. 10.3389/fpls.2019.01134 31611888 PMC6776121

[B54] Moon H. Lee H. Paek K. Park S. (2015). Osmotic stress and strong 2,4-D shock stimulate somatic-to-embryogenic transition in *Kalopanax septemlobus* (Thunb.) Koidz. *Acta Physiol. Plant* 37:1710. 10.1007/s11738-014-1710-x

[B55] Mortazavi A. Williams B. A. Mccue K. Schaeffer L. Wold B. (2008). Mapping and quantifying mammalian transcriptomes by RNA-Seq. *Nat Methods* 5 621–628. 18516045 10.1038/nmeth.1226 PMC13303166

[B56] Narsai R. Secco D. Schultz M. D. Ecker J. R. Lister R. Whelan J. (2017). Dynamic and rapid changes in the transcriptome and epigenome during germination and in developing rice (Oryza sativa) coleoptiles under anoxia and re-oxygenation. *Plant J.* 89 f805–f824. 10.1111/tpj.13418 27859855

[B57] Nascimento W. M. Huber D. J. Cantliffe D. J. (2013). Carrot seed germination and respiration at high temperature in response to seed maturity and priming. *Seed Sci. Technol.* 41 164–169.

[B58] Nguyen H. M. Sako K. Matsui A. Suzuki Y. Mostofa M. G. Ha C. V. (2017). Ethanol enhances high-salinity stress tolerance by detoxifying reactive oxygen species in arabidopsis thaliana and rice. *Front. Plant Sci.* 8:1001. 10.3389/fpls.2017.01001 28717360 PMC5494288

[B59] Nogales A. Muñoz-Sanhueza L. Hansen L. D. Arnholdt-Schmitt B. (2015). Phenotyping carrot (*Daucus carota* L.) for yield-determining temperature response by calorespirometry. *Planta* 241 525–538. 10.1007/s00425-014-2195-y 25380771

[B60] Nogales A. Nobre T. Cardoso H. G. Muñoz-Sanhueza L. Valadas V. Campos M. D. (2016). Allelic variation on DcAOX1 gene in carrot (*Daucus carota* L.): an interesting simple sequence repeat in a highly variable intron. *Plant Gene* 5 49–55. 10.1016/j.plgene.2015.11.001

[B61] Paszkiewicz G. Gualberto J. M. Benamar A. Macherel D. Logan D. C. (2017). Arabidopsis seed mitochondria are bioenergetically active immediately upon imbibition and specialize via biogenesis in preparation for autotrophic growth. *Plant Cell* 29 109–128. 10.1105/tpc.16.00700 28062752 PMC5304351

[B62] Pengpeng J. Chenyu D. Penghu C. Dong S. Ruizhuo O. Yuqing M. (2020). The role of reactive oxygen species in tumor treatment. *RSC Adv.* 10 7740–7750. 10.1039/c9ra10539e PMC9049915 35492191

[B63] Porfirio S. Calado M. L. Noceda C. Cabrita M. J. da Silva M. G. Azadi P. (2016). Tracking biochemical changes during adventitious root formation in olive (Olea europaea L.). *Sci. Hort.* 204 41–53. 10.1016/j.scienta.2016.03.029

[B64] Powell A. A. (2017). *A review of the Principles and Use of the Q2 Seed Analyser.* Aberdeen: International Seed Testing Association.

[B65] Purushothaman R. Upadhyaya H. D. Gaur P. M. Gowda C. L. L. Krishnamurthy L. (2014). Kabuli and desi chickpeas differ in their requirement for reproductive duration. *Field Crops Res.* 163 24–31. 10.1016/j.fcr.2014.04.006

[B66] Qi W. Ma L. Wang F. Wang P. Wu J. Jin J. (2020). Reactive oxygen species as important regulators of cell division. *Biorxiv.* [Preprint] 10.1101/2020.03.06.980474

[B67] European Parliament and of the Council. (2018). *Regulation (EU) 2018/848 of the European Parliament and of the Council of 30 May 2018 on organic production and labelling of organic products and repealing Council Regulation (EC) No 834/2007.* Brussels: European Parliament and of the Council.

[B68] Sakr S. Wang M. Dédaldéchamp F. Perez-Garcia M. D. Ogé L. Hamama L. (2018). The Sugar-Signaling Hub: Overview of Regulators and Interaction with the Hormonal and Metabolic Network. *Int. J. Mol. Sci.* 19:2506. 10.3390/ijms19092506 30149541 PMC6165531

[B69] Saleh T. Kalodimos C. G. (2017). Enzymes at work are enzymes in motion. *Science* 355 247–248. 10.1126/science.aal4632 28104853

[B70] Sangüesa G. Roglans N. Baena M. Velázquez A. M. Laguna J. C. Alegret M. (2019). mTOR is a Key Protein Involved in the Metabolic Effects of Simple Sugars. *Int. J. Mol. Sci.* 20:1117. 10.3390/ijms20051117 30841536 PMC6429387

[B71] Santos Macedo E. Cardoso H. G. Hernández A. Peixe A. A. Polidoros A. Ferreira A. (2009). Physiologic responses and gene diversity indicate olive alternative oxidase as a potential source for markers involved in efficient adventitious root induction. *Physiol. Plant* 137 532–552. 10.1111/j.1399-3054.2009.01302.x 19941624

[B72] Saraiva K. D. Oliveira A. E. Santos C. P. Lima K. T. Sousa J. M. Melo D. F. (2016). Phylogenetic analysis and differential expression of EF1α genes in soybean during development, stress and phytohormone treatments. *Mol. Genet. Genom.* 291 1505–1522. 10.1007/s00438-016-1198-8 26984342

[B73] Schmidt R. R. Weits D. A. Feulner C. F. J. van Dongen J. T. (2018). Oxygen sensing and integrative stress signaling in plants. *Plant Physiol.* 176 1131–1142. 10.1104/pp.17.01394 29162635 PMC5813526

[B74] Selinski J. Hartmann A. Deckers-Hebestreit G. Day D. A. Whelan J. Scheibe R. (2018). Alternative oxidase isoforms are differentially activated by tricarboxylic acid cycle intermediates. *Plant Physiol.* 176 1423–1432. 10.1104/pp.17.01331 29208641 PMC5813554

[B75] Siegień I. Bogatek R. (2006). Cyanide action in plants — from toxic to regulatory. *Acta Physiol. Plant.* 28 483–497.

[B76] Simon P. W. Navazio J. P. Colley M. McCluskey C. Zystro J. Hoagland L. (2017). The CIOA (carrot improvement for organic agriculture) project: location, cropping system and genetic background influence carrot performance including top height and flavour. *Acta Horticulturae* 1153 1–8. 10.17660/ActaHortic.2017.1153.1

[B77] Sircar D. Cardoso H. G. Mukherjee C. Mitra A. Arnholdt-Schmitt B. (2012). Alternative oxidase (AOX) and phenolic metabolism in methyl jasmonate-treated hairy root cultures of Daucus carota L. *J Plant Physiol.* 169 657–663. 10.1016/j.jplph.2011.11.019 22326792

[B78] Sivasakthi K. Tharanya M. Kholová J Wangari Muriuki R. Thirunalasundari T. Vadez V. (2017). Chickpea genotypes contrasting for vigor and canopy conductance also differ in their dependence on different water transport pathways. *Front Plant Sci.* 8:1663. 10.3389/fpls.2017.01663 29085377 PMC5649140

[B79] Srivastava S. Conlan X. A. Cahill D. M. Adholeya A. (2016). Rhizophagus irregularis as an elicitor of rosmarinic acid and antioxidant production by transformed roots of Ocimum basilicum in an in vitro co-culture system. *Mycorrhiza* 26 919–930. 27485855 10.1007/s00572-016-0721-4

[B80] Vanlerberghe G. C. Vanlerberghe A. E. McIntosh L. (1994). Molecular genetic alteration of plant respiration (silencing and overexpression of alternative oxidase in transgenic tobacco). *Plant Physiol.* 106 f1503–f1510. 10.1104/pp.106.4.1503 12232424 PMC159691

[B81] Vicente C. Costa J. H. Arnholdt-Schmitt B. (2015). “Bacterial AOX: a provocative lack of interest!,” in *Alternative Respiratory Pathways in Higher Plants*, eds Gupta K. J. Mur L. A. Neelwarne B. (Hoboken, NJ: Wiley Publishing group), 319–322.

[B82] Vicente S. L. C. Arnholdt-Schmitt B. (2008). “Characterization of mediterranean AMs: initiation of a novel functional marker approach,” in *Proceedings of the COST 870 Meeting*, Greece.

[B83] Voss-Fels K. P. Cooper M. Hayes B. J. (2019). Accelerating crop genetic gains with genomic selection. *Theor. Appl. Genet.* 132 669–686. 10.1007/s00122-018-3270-8 30569365

[B84] Wang W. R. Liang J. H. Wang G. F. Sun M. X. Peng F. T. Xiao Y. S. (2020). Overexpression of PpSnRK1α in tomato enhanced salt tolerance by regulating ABA signaling pathway and reactive oxygen metabolism. *BMC Plant Biol.* 20:128. 10.1186/s12870-020-02342-2 32216751 PMC7099830

[B85] Wassermann B. Cernava T. Müller H. Berg C. Berg G. (2019). Seeds of native alpine plants host unique microbial communities embedded in cross-kingdom networks. *Microbiome* 7:108. 10.1186/s40168-019-0723-5 31340847 PMC6651914

[B86] Wojtyla Ł Lechowska K. Kubala S. Garnczarska M. (2016). Different modes of hydrogen peroxide action during seed germination. *Front. Plant Sci.* 7:66. 10.3389/fpls.2016.00066 26870076 PMC4740362

[B87] Wurzinger B. Nukarinen E. Nägele T. Weckwerth W. Teige M. (2018). The SnRK1 kinase as central mediator of energy signaling between different organelles. *Plant Physiol.* 176 1085–1094. 10.1104/pp.17.01404 29311271 PMC5813556

[B88] Zabalza A. van Dongen J. T. Froehlich A. Oliver S. N. Faix B. Gupta K. J. (2009). Regulation of respiration and fermentation to control the plant internal oxygen concentration. *Plant Physiol.* 149 1087–1098. 10.1104/pp.108.129288 19098094 PMC2633817

[B89] Zavattieri M. A. Frederico A. M. Lima M. Sabino R. Arnholdt-Schmitt B. (2010). Induction of somatic embryogenesis as an example of stress-related plant reactions. *J. Biotechnol.* 13:1. 10.2225/vol13-issue1-fulltext-4

[B90] Zhao J. Li W. Sun S. Peng L. Huang Z. He Y. (2021). The rice small auxin-up rna gene ossaur33 regulates seed vigor via sugar pathway during early seed germination. *Int. J. Mol. Sci.* 22:1562. 10.3390/ijms22041562 33557166 PMC7913900

[B91] Zhu T. Zou L. Li Y. Yao X. Xu F. Deng X. (2018). Mitochondrial alternative oxidase-dependent autophagy involved in ethylene-mediated drought tolerance in *Solanum lycopersicum*. *Plant Biotechnol. J.* 16 2063–2076. 10.1111/pbi.12939 29729068 PMC6230944

