# Supplementary material for: Adaptive Reprogramming During Early Seed Germination Requires Temporarily Enhanced Fermentation-A Critical Role for Alternative Oxidase Regulation That Concerns Also Microbiota Effectiveness
Source: Front Plant Sci. 2021 Oct 1;12:686274. doi: 10.3389/fpls.2021.686274 (PMC8518632; doi:10.3389/fpls.2021.686274)
Supplement: Supplementary Figure 1 — Exogenous sucrose delayed callus emergence and was necessary for SE. [file Data_Sheet_1.zip › New folder (2)/Figure 2.DOCX]

**Supplementary Figure S2**:

**Figure S2**: 2 h pulse with commercial sugar improved carrot germination efficiency monitored at 40 HAI and 50 HAI

**

**
