# Supplementary material for: Adaptive Reprogramming During Early Seed Germination Requires Temporarily Enhanced Fermentation-A Critical Role for Alternative Oxidase Regulation That Concerns Also Microbiota Effectiveness
Source: Front Plant Sci. 2021 Oct 1;12:686274. doi: 10.3389/fpls.2021.686274 (PMC8518632; doi:10.3389/fpls.2021.686274)
Supplement: Supplementary Figure 1 — Exogenous sucrose delayed callus emergence and was necessary for SE. [file Data_Sheet_1.zip › New folder (2)/Figure 3.DOCX]

**Supplementary Figure S3**:

**Figure S3**: Effect of SHAM treatment on accumulation of soluble and wall bound phenolics (A) and flavonoids and lignin (B) in elicitor-treated hairy roots of *Daucus carota*. Values obtained in only elicitor-treated root was considered as 100% and results were expressed in terms of percentage of maximum. The terms E and NE in the x-axis legend denote -with and -without elicitor, respectively. * Soluble phenolics. Values are mean of three independent experiments ± SD.

**% of maximum**

**A**

**B**
