# Supplementary material for: Adaptive Reprogramming During Early Seed Germination Requires Temporarily Enhanced Fermentation-A Critical Role for Alternative Oxidase Regulation That Concerns Also Microbiota Effectiveness
Source: Front Plant Sci. 2021 Oct 1;12:686274. doi: 10.3389/fpls.2021.686274 (PMC8518632; doi:10.3389/fpls.2021.686274)

**Supplementary Figure S4:**

**Figure S4: Rapid germination check of organic and conventional seeds from seven cultivars in water (control) or under SHAM (5 mM) treatment**


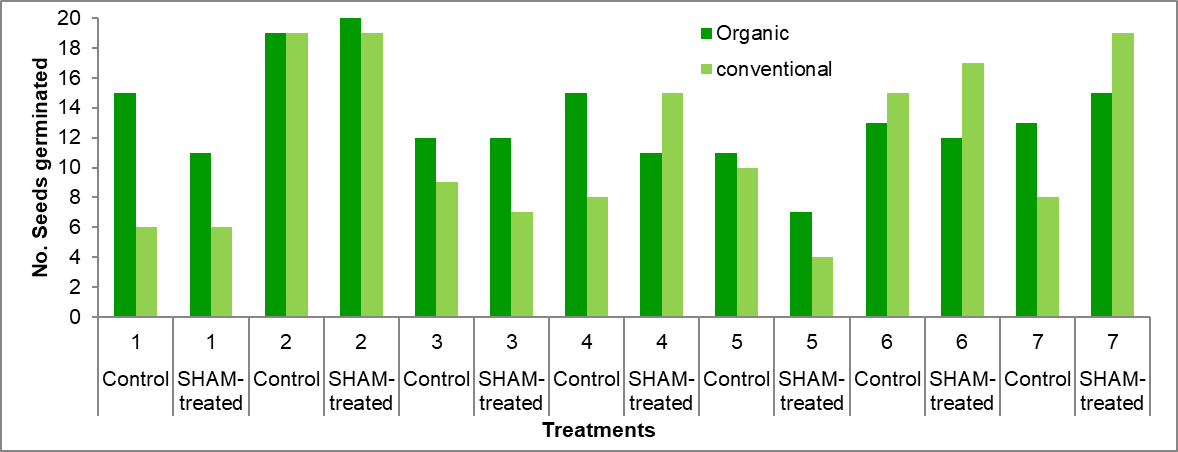

Supplement: Supplementary Figure 1 — Exogenous sucrose delayed callus emergence and was necessary for SE. [file Data_Sheet_1.zip › New folder (2)/Figure 4.DOCX]
