# Supplementary material for: Adaptive Reprogramming During Early Seed Germination Requires Temporarily Enhanced Fermentation-A Critical Role for Alternative Oxidase Regulation That Concerns Also Microbiota Effectiveness
Source: Front Plant Sci. 2021 Oct 1;12:686274. doi: 10.3389/fpls.2021.686274 (PMC8518632; doi:10.3389/fpls.2021.686274)
Supplement: Supplementary Figure 1 — Exogenous sucrose delayed callus emergence and was necessary for SE. [file Data_Sheet_1.zip › New folder (2)/Table 1 and Figure 1.DOCX]

|  | Callus induction efficiency  (in %) | |
| --- | --- | --- |
| Con. of Sucrose | SSS | EFS |
| 0% | 0±0 | 0±0 |
| 0.5% | 0±0 | 0±0 |
| 2% | 17±1.4 | 21.5±0.7 |
| 3% | 33.5±3.5* | 38±2.8* |
| 7% | 0±0 | 0±0 |
| p value | 0.025 | 0.0150 |

**Supplementary Figure 1:** Exogenous sucrose delayed callus emergence and was necessary for SE

c

c

c

b

a

b

b

b

b

a

c

bc

bc

a

a

c

bc

bc

b

a

c

c

b

b

a

d

d

c

b

a

d

d

c

b

a

c

a

b

b

ab

ab

b

ab

ab

The surface sterilized seeds used for SE callus inducton at different concentration of sucrose. Suc. – Sucrose; DAI – Days after inoculation; The bar represent the mean ± SD; Tukey test at the P>0.05.

**Supplementary Table S1:** Effect of exogenous sucrose concentration on carrot SE callus induction

|  | Callus induction efficiency  (in %) | |
| --- | --- | --- |
| Con. of Sucrose | Surface sterlized seeds | Endophyte free seeds |
| 0% | 0±0 | 0±0 |
| 0.5% | 0±0 | 0±0 |
| 2% | 17±1.4 | 21.5±0.7 |
| 3% | 33.5±3.5* | 38±2.8* |
| 7% | 0±0 | 0±0 |
| *p* value | 0.025 | 0.0150 |

SSS – Surface sterilized seeds; EFS – Endophytes free seeds, % - percentage; Values represent the mean ± SD; * indicates statistically significant (Student’s T – Test). The *p* value was compared between 2% and 3% sucrose on SE callus induction efficiency.
